# Supplementary material for: Xenorhabdus and Photorhabdus Metabolites for Fungal Biocontrol and Application in Soybean Seed Protection
Source: J Fungi (Basel). 2025 Sep 23;11(10):691. doi: 10.3390/jof11100691 (PMC12565595; doi:10.3390/jof11100691)
Supplement: Supplementary file 1 [file jof-11-00691-s001.zip › jof-3847589-supplementary.pdf]

**Table S1.** Symbiotic bacteria evaluated with their respective entomopathogenic nematodes.

| **Symbiotic Bacteria** | **Nematode** | **Strain** |
|---|---|---|
| *Xenorhabdus szentirmaii* | *Steinernema rarum* | PAM 25 |
| *X. szentirmaii* | *S. rarum* | DSM 16338 |
| *X. nematophila* | *S. carpocapsae* | ATCC 19061 |
| *X. doucetiae* | *S. diaprepesi* | DSM 17909 |
| *Photorhabdus kayaii* | *Heterorhabditis bacteriophora* | DSM 15194 |
